# Characterization of Human Papillomavirus prevalence and risk factors to guide cervical cancer screening in the North Tongu District, Ghana

**DOI:** 10.1371/journal.pone.0218762

**Published:** 2019-06-27

**Authors:** Amrei Krings, Priscilla Dunyo, Aleksandra Pesic, Saviour Tetteh, Benjamin Hansen, Isaac Gedzah, Comfort M. Wormenor, Joseph E. Amuah, Anna-Lisa Behnke, Daniela Höfler, Michael Pawlita, Andreas M. Kaufmann

**Affiliations:** 1 Clinic for Gynecology, Laboratory for Gynecologic Tumor Immunology, Charité - Universitätsmedizin Berlin, Corporate Member of Freie Universität Berlin, Humboldt-Universität zu Berlin, and Berlin Institute of Health, Campus Benjamin Franklin, Berlin, Germany; 2 Catholic Hospital Battor, Battor, Volta Region, Ghana; 3 Department of Epidemiology and Community Medicine, University of Ottawa, Ottawa, Canada; 4 Division of Molecular Diagnostics of Oncogenic Infections, Research Program Infection, Inflammation and Cancer, German Cancer Research Center (DKFZ), Heidelberg, Germany; Greenebaum Cancer Center, Institute of Human Virology, University of Maryland School of Medicine, UNITED STATES

## Abstract

**Introduction:**

This population-based study aimed to fill the knowledge gap on Human Papillomavirus (HPV) prevalence and associated sociodemographic risk factors of the general population in the North Tongu District, Ghana. These results are needed to guide cervical cancer prevention efforts, as the leading type of female cancers.

**Methods:**

A cross-sectional study including 2002 women in the North Tongu District, Ghana investigated HPV prevalence and associated sociodemographic risk factors. Women were recruited by geographical distribution through the local community-based health system and samples collected using a self-sampling device. For HPV genotyping BSGP5+/6+-PCR with Luminex-MPG readout was used. Multivariate logistic regression analyzed sociodemographic risk factors for HPV positivity.

**Results:**

Of 2002 self-collected samples, 1943 were eligible, contained sufficient DNA and provided valid HPV genotyping results. Prevalence of single high risk HPV types was 32.3% and of multiple high risk types 9.7%. The five most common detected HPV types were HPV16 (7.4%; 95%CI: 6.3–8.7), HPV52 (7.2%; 95%CI: 6.1–8.5), HPV35 (4.8%; 95%CI: 3.9–5.8), HPV59 (4.7%; 95%CI: 3.8–5.8), HPV56 (3.9%; 95%CI: 3.1–4.8). Highest prevalence was observed among women aged 18–24 years, while age 25–54 years was inversely associated with high risk HPV positivity in multivariate analysis. Sociodemographic risk factors identified were i) having any sexual partner, ii) more partners increased the odds for high risk HPV positivity, iii) independently from this marital status, in particular not being married.

**Discussion & conclusion:**

Most importantly, the high risk HPV prevalence detected from this study is higher than estimates reported for Western Africa. This needs be considered, when deciding on the cervical cancer screening algorithms introduced on a wider scale. Follow-up and triage, depending on the methods chosen, can easily overburden the health system. Self-sampling worked well and provided adequate samples for HPV-based screening. Women with increasing number of sexual partners and not being married were found to have higher odds of being high risk HPV positive, therefore could be a higher prioritized screening target group.

## Introduction

### Global situation HPV & cervical cancer

Cervical cancer is the 4th most common cancer among women in the world leading to about 528.000 registered new cancer cases and 266.000 cervical cancer deaths annually [1]. About 99% of cervical cancer cases are caused by persistent infection with Human Papillomaviruses (HPV) [2] and genotypes HPV16 and HPV18 cause approx. 70% of the global cervical cancer cases [3]. Regional differences exist for the HPV genotype distribution in cervical cancer. In Sub-Saharan Africa HPV16, 18, 45, 35 and 33 were identified as the most common types [4], while globally HPV16, 18, 45, 33 and 31 are most common in the respective order [3]. With different risks for and time to cancer progression as well as possible protection from HPV vaccination, genotype prevalence and distribution are important factors to investigate in each world region.

Low-middle income countries (LMIC) carry the greatest global burden of cervical cancer with about 85% of incident cases and 87% of annual deaths occurring there [1]. Reasons for this are late presentation at the health facilities [5], poorly developed health systems, lack of financial and technical resources as well as human capacity to diagnose and treat cervical cancer, and often also lack of awareness [6]. This high rate of cervical cancer incidence is projected to increase by 90% until 2030 when considering the current increase in incidence as well as aging and population growth [7]. Factors such as the increasing number of HIV-positive patients further contribute to this projection [7]. While HPV vaccines are available and provide effective primary prevention, their accessibility in LMICs is still limited mostly to initiatives by the Global Alliance for Vaccines and Immunization (GAVI). Furthermore, the available vaccines do not cover all high risk HPV types and therefore the need for simple, affordable and acceptable secondary prevention remains inevitable. A review of policy documents and interviews with key stakeholders from Tanzania, Kenya and Uganda shows that although the advantage of HPV testing for cervical cancer screening is understood, screening remains rare and is often offered to women outside the recommended age range. Programs are underfunded, resulting in low coverage and insufficient quality [8].

In Ghana cervical cancer is the 2nd most common female cancer among women at the age of 15 to 44 years and the 2nd leading cause of cancer deaths [9]. With a crude incidence rate of 18.6 per 100.000 annually, cervical cancer is even more common in Ghana compared to the Western Africa region with 16.8 per 100.000 [1, 9]. At the Korle Bu Teaching Hospital Accra 57.8% of the women presenting with gynecological cancer had cervical cancer [10].

Despite this high burden of disease little is known about the prevalence of HPV infection and its risk factors in the general population of Ghana. Studies investigating this are very rare and limited to small studies (n<230) or specialized populations (e.g. referral population, cervical cancer patients, pregnant women). Prevalence rates stated are for example 10.7% among 75 women attending the gynecology outpatient clinic in Accra [11], or 42% and 76.6% among 100 HIV negative and 107 HIV positive women from Kumasi, respectively [12]. A study among pregnant women from the Western Region of Ghana detected 13.9% of the women to be high risk HPV positive [13]. Few studies focused on the HPV genotype distribution among cervical cancer patients. The Pathology Department at the University of Ghana found the most common HPV types among 230 cervical cancer patients to be HPV18 (47.4%), HPV59 (42.2%), HPV45 (37.4%) and HPV16 (9.0%) with 52.2% having multiple HPV types [14]. Thus, no large population-based study depicting the HPV prevalence has been published from Ghana to date.

### The ACCESSING study

ACCESSING is an acronym for “*A*dequate *C*ervical cancer *C*apacity building, *E*ducation and *S*creening by new *S*cientific *IN*struments in *G*hana” and a program funded by the Deutsche Gesellschaft für Internationale Zusammenarbeit (GIZ) and the German Rotary Voluntary Doctors (GRVD). The program was conducted during a hospital partnership in collaboration between the Catholic Hospital Battor in the Volta Region, Ghana, and the Charité—Universitätsmedizin Berlin, Clinic for Gynecology, Germany. The aims of this program were to assess the HPV and STI prevalence with a cross-sectional study design in the general population, to evaluate a potential screening algorithm for cervical cancer screening on its feasibility, and to build the capacity to independently and autonomously introduce cervical cancer screening. The program was conducted from October 2013 until February 2017 including two pilot studies with 250 and 150 women screened at the Catholic Hospital Battor, respectively, and a main study with 2002 women screened from the rural and urban communities in the North Tongu District.

This manuscript presents the results from the ACCESSING main study investigating HPV prevalence, genotype distribution, and the sociodemographic risk factors associated with high risk HPV positivity. This shall help decision makers to identify adequate cervical cancer screening methods and potentially prioritized risk groups for screening.

## Methods

### Study population

This evaluation of the ACCESSING program is a cross-sectional study conducted in the North Tongu District, Ghana in a collaboration of the Catholic Hospital Battor, Ghana and the Charité—Universitätsmedizin Berlin, Germany. Women in the age of 18 to 65 years without a history of cervical cancer and who were not pregnant were recruited.

Sample size calculation was based on a HPV prevalence estimate of 21.3% and the objective to identify a clinically relevant difference of 2.5% to this suggested prevalence [15]. Adjusting for a potential loss to follow-up, loss of sample material or unusable samples of between 5–6% and at a 5% significance level with 80% statistical power, the sample size needed was 2003 [16]. Thus a sample size of 2000 women was proposed to be sufficient to detect a clinically important difference.

Ethical clearance for this study was given by the Ghana Health Service Ethical Review Committee (Ref. No. GHD-ERC: 05/05/13) in October 2013. Signed/thumb-printed written/translated informed consent was obtained from all women participating in the screening.

### Sample collection

The number of women included in each village within the North Tongu District was defined based on the population size reported during the latest Population Census from 2010 and adjusted to a sample size of 2000 study participants [17]. Recruitment of study participants was conducted by Community Health Workers (CHW), as part of the Ghana Health Service Community-based Health Planning and Services (CHPS), who are based in the respective villages, on a door-to-door and first-come-first-serve basis.

The samples were collected using an approved (Ghana FDA) self-sampling device, namely the Evalyn brush (Rovers Medical Devices, Oss, The Netherlands). Evalyn Brush samples were self-collected or collected with assistance from a CHW according to manufacturer’s instructions and stored dry and at ambient temperature for a maximum time period of 7 days until arrival in the laboratory of the Catholic Hospital Battor, Ghana. Every woman screened filled out a questionnaire asking for general demographic data (e.g. age, education, and income level per month) as well as specific risk factors such as age at first intercourse, number of sexual partners, etc. (see supporting information S1 File for the questionnaire).

### Sample processing & HPV genotyping

The brush head was removed from the self-sampling applicator into a 2 ml Eppendorf tube and 1 ml of PreservCyt solution was added and vortexed vigorously for about 1 min. After incubation overnight at room temperature brushes were vortexed again and then removed. 100 μl from this cell suspension were aliquoted and stored at -20°C for DNA extraction at the Laboratory for Gynecologic Tumor Immunology, Clinic for Gynecology, Charité Universitätsmedizin Berlin.

For DNA extraction, the Maxwell 16 LEV Blood DNA Kit (Promega GmbH, Mannheim, Germany) was used according to manufacturer’s instructions. DNA was eluted into 60 μl of elution buffer and stored at -20°C until further used.

Genotyping of mucosal HPV was done by BSGP5+/6+ PCR followed by Luminex-MPG read-out according to Schmitt et al. 2008 [18] detecting the genotypes 6, 11, 16, 18, 26, 31, 33, 35, 39, 42, 43, 45, 51, 52, 53, 54, 56, 57, 58, 59, 66, 68a, 68b, 70, 72, 73, 82 and 90 at Charité—Universitätsmedizin Berlin, Germany. Clinical performance of this test has been validated and considered useful for HPV-based cervical cancer screening [19]. Results were considered valid if sufficient DNA was present, indicated by a Luminex measured Median Fluorescent Intensity (MFI) of 200 or more for ß-globin, which was used as a proxy for cellular material. The MFI is interpreted qualitatively, indicating if a sample is positive or negative for the respective HPV type, but not used for quantitative information on the viral load of samples.

According to WHO monograph classification HPV types 16, 18, 31, 33, 35, 39, 45, 51, 52, 56, 58, and 59 were considered as high risk HPV genotypes. HPV66 and 68a and 68b were defined as probable, HPV26, 53, 73, and 82 as potential high risk and the HPV types 6, 11, 42, 43, 54, 57, 70, 72, 90 as low risk types [20].

### Clinical follow-up

Women who tested positive for high risk HPV were recalled to the clinic for cytology and upon abnormal cytology result called again for colposcopy. Based on the diagnosis by the treating gynecologist treatment, such as loop electrosurgical excision procedure (LEEP) or hysterectomy, was provided. Cytological examination was done at the Department of Pathology, University of Cape Coast, Ghana. HPV testing as well as any clinical follow-up required were provided free of charge for the study participants.

### Statistical analysis

Statistical analysis of categorical data was done using high risk HPV positivity as the dependent variable. Univariate and multivariate logistical regression analysis was used to identify potential sociodemographic risk factors for high risk HPV positivity. Variables for multivariate logistic regression were chosen based on their association resulting from univariate analysis as well as from sociodemographic risk factors reported in the literature. The multivariate model was built using forward stepwise selection and the likelihood ratio test to compare the fit of the models. The unadjusted odds ratio (OR), adjusted odds ratio (AOR), 95% confidence intervals (95%CI) and p-values from univariate and multivariate logistic regression were reported as a measure of association. All reported p-values were 2-sided with a significance level of 0.05. For statistical analysis STATA version 15 (StataCorp LLC, College Station, Texas, USA) was used.

## Results

**Fig 1:**
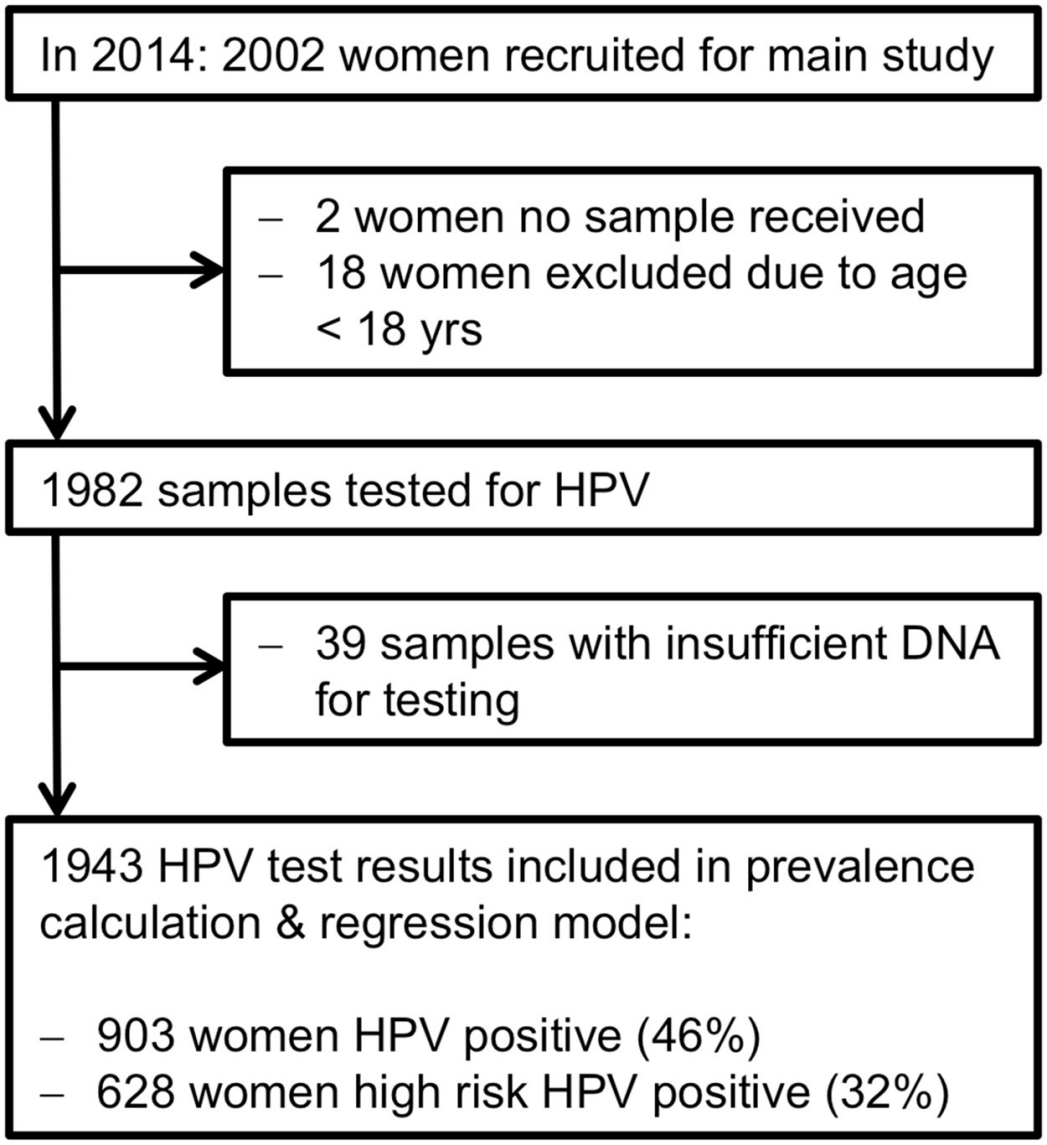
Flow chart of ACCESSING main HPV screening study in 2014.

From a total of 2002 women samples with a self-sampling brush were collected through the CHW system in the North Tongu District within 5 weeks. Two samples did not reach the laboratory, 18 were excluded from this analysis because they were not within the age range of 18–65 years and an additional 39 samples were excluded due to insufficient DNA present in the sample for HPV testing. Hence a total of 1943 samples were included in the HPV and risk factor analysis (Fig 1). Among the 2002 samples tested this represents sufficient DNA for 98% of samples.

### Sociodemographic description

The distribution of sociodemographic characteristics is summarized in Table 1 with a few details described here: The average age of the women tested was 32 years of age. The majority of women completed Junior High School or higher (59.8%) and about half of the women had a monthly income of less than 100 Ghana Cedi (GHS), which calculated to approx. US $ 25 per month (in August 2015). The most common occupations were trading and farming and about 9.5% were students or doing an apprenticeship. Many women recruited were married (42.2%) and had 1–2 children (38.0%), ranging to up to 13 children. The majority did not use any contraceptive (66.0%) and had 2–3 sexual partners. Almost half (48.0%) of the women were between 15–18 years when they had their first sexual intercourse.

**Table 1:** Sociodemographic and behavioral characteristics of eligible study participants (n = 1982).

Sociodemographic characteristics	n	Prevalence in %
**Age**		
Mean: 31.9 years		
18–24	552	27.9
25–34	744	37.5
35–44	403	20.3
45–54	205	10.3
55–65	78	3.9
**Education**		
None	346	17.5
Primary	436	22.0
Junior High School	793	40.0
Secondary	254	12.8
Post Secondary	73	3.7
Post Graduate	65	3.3
missing data	15	0.8
**Income level per month**		
<100 GH¢	989	49.9
100–250 GH¢	329	16.6
251–500 GH¢	106	5.4
>500 GH¢	109	5.5
missing data	449	22.7
**Occupation**		
Apprentice/Student	188	9.5
Farmer	494	24.9
Hairdresser	82	4.1
Worker in Health care sector	34	1.7
Seamstress	112	5.7
Teacher	74	3.7
Trader	668	33.7
Unemployed/House wife/Retired	129	6.5
Food vendor	46	2.3
Office Employee	30	1.5
Cleaner	9	0.5
missing data	116	5.9
**Marital Status**		
Single	178	9.0
Have a steady partner	395	19.9
Living with someone (unmarried)	428	21.6
Married	836	42.2
Divorced	86	4.3
Widowed	58	2.9
missing data	1	0.1
**Number of sexual partners**		
None	23	1.2
1	760	38.4
2–3	964	48.6
>3	210	10.6
missing data	25	1.3
**Number of children**		
None	355	17.9
1–2	754	38.0
3–4	483	24.4
5–6	247	12.5
>6	138	7.0
missing data	5	0.3
**Age at first intercourse**		
<15	66	3.3
15–18	951	48.0
19–22	628	31.7
23–26	103	5.2
>26	24	1.2
missing data	210	10.6
**Contraceptive Use**		
None	1308	66.0
Injectable	347	17.5
Pill	140	7.1
Norplant/Jadelle	82	4.1
Condom	65	3.3
Abstinence	24	1.2
IUCD	4	0.2
Other	8	0.4
missing data	4	0.2
**Current smoking**		
Yes	19	1.0
No	1953	98.5
missing data	10	0.5

Abbreviations: GH¢—Ghana Cedi (100 GH¢ ~ 25US$, exchange rate in August 2015); IUCD—Intrauterine contraceptive device

### HPV prevalence results from the North Tongu District, Ghana

Among the 1943 samples left for analysis, high risk HPV was detected in 32.3% (95%CI: 30.2–34.5) and 9.7% (95%CI: 8.4–11.1) were positive for multiple high risk HPV types. On average women with multiple high risk types had 1.4 high risk types detected (range: 1–7 high risk types). 18.4% (95%CI: 17.7–20.2) were positive for low risk HPV as single or multiple infection and 53.5% (95%CI: 51.3–55.8) HPV negative. Among women aged 30–49 years, the WHO recommended screening age, high risk HPV prevalence was 27.3% (95%CI: 24.3–30.5). Women high risk HPV positive were invited for clinical follow up free of charge. This was structured in cytology and based on indication colposcopy, LEEP and potentially hysterectomy. Results of clinical follow up are presented in the supporting information S1 Fig.

The five most prevalent high risk HPV types were HPV16 (7.4%; 95% CI: 6.3–8.7), HPV52 (7.2%; 95% CI: 6.1–8.5), HPV35 (4.8%; 95%CI: 3.9–5.8), HPV59 (4.7% 95% CI: 3.8–5.8) and HPV56 (3.9%; 95% CI: 3.1–4.8) (Table 2). HPV66, as a probable high risk type was detected in 4.3% (95% CI: 3.5–5.3) of the women. The most common low risk type detected was HPV42 (7.1%; 95% CI: 6.0–8.3).

**Table 2:** Prevalence of HPV by genotype among eligible women (n = 1943).

**HPV Type positivity**	**Infections positive (n)**	**(%)**	**95% CI**
High risk HPV			
16	144	7.4	6.3–8.7
18	72	3.8	2.9–4.6
31	47	2.4	1.8–3.2
33	15	0.8	0.4–1.3
35	93	4.8	3.9–5.8
39	40	2.1	1.5–2.8
45	63	3.2	2.5–4.1
51	58	3.0	2.3–3.8
52	140	7.2	6.1–8.5
56	75	3.9	3.1–4.8
58	45	2.3	1.7–3.1
59	92	4.7	3.8–5.8
Probable high risk HPV			
66	84	4.3	3.5–5.3
68a	28	1.4	1.0–2.1
68b	47	2.4	1.8–3.2
Potential high risk HPV			
26	7	0.4	0.1–0.7
53	72	3.7	2.9–4.6
73	24	1.2	0.8–1.8
82	59	3.0	2.3–3.9
Low risk HPV			
6	51	2.6	2.0–3.4
11	12	0.6	0.3–1.1
42	137	7.1	6.0–8.3
43	23	1.2	0.8–1.8
54	50	2.6	1.9–3.4
57	1	0.1	<0.1–0.3
70	45	2.3	1.7–3.1
72	12	0.6	0.3–1.1
90	87	4.5	3.6–5.5
**HPV positivity**	**women positive (n)**	**(%)**	**95% CI**
HPV negative	1040	53.5	51.3–55.8
High risk HPV+	628	32.3	30.2–34.5
Single high risk HPV+	440	22.6	20.8–24.6
Multiple high risk HPV+	188	9.7	8.4–11.1
High risk AND probable HPV+	697	35.9	33.7–38.1
High risk AND probable AND potential HPV+	750	38.6	36.4–40.8
Low risk HPV+	405	18.4	17.7–20.2

Abbreviations: OR—odds ratio; CI—Confidence interval; HPV—Human Papillomavirus

**Fig 2:**
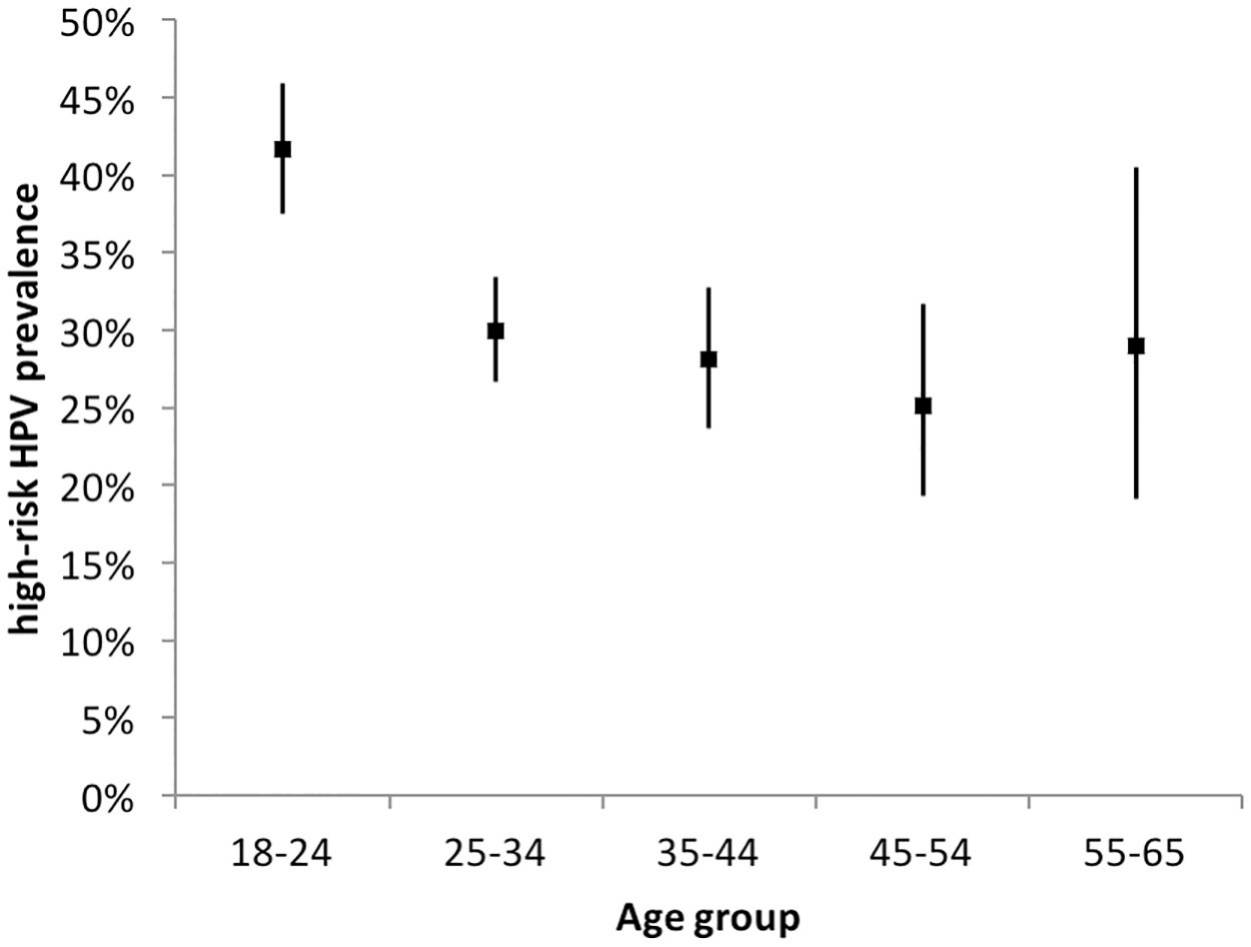
High-risk HPV prevalence by age group.

The age-specific HPV prevalence is presented in Fig 2 and was highest among women younger than 25 years (41.7%; 95% CI: 37.5–45.9). With increasing age, prevalence decreases up to 54 years and then increases again in the oldest age group of 55–65 years. Lowest prevalence is seen in the age group 45–54 years with 25.1% (95% CI: 19.3–31.7).

### Risk factors associated with high risk HPV positivity

Univariate analysis suggests high evidence for decreasing odds ratio (OR) of high risk HPV positivity for women with the following sociodemographic factors (Table 3). Women at older age had lower ORs for high risk HPV infection compared to those of younger age. Furthermore, women who work as farmers (OR: 0.4; 95%CI: 0.3–0.6; p-value: <0.001), teacher (OR: 0.5, 95%CI: 0.3–0.8; p-value: 0.010), traders (OR: 0.6; 95%CI: 0.4–0.8; p-value: 0.001) or in the health care sector (OR: 0.4; 95%CI: 0.2–0.9; p-value: 0.032) were associated with a decreased OR for infection compared to students or women doing an apprenticeship. The OR increased for women who completed primary (OR: 1.5; 95%CI: 1.1–2.0; p-value: 0.016) or secondary school (OR: 1.7; 95%CI: 1.2–2.5; p-value: 0.002) in comparison to those without any educational degree. No such association was seen for women who completed Junior High School. High evidence for increasing ORs of high risk HPV positivity was seen among women who are single (OR: 2.6; 95%CI: 1.8–3.6; p-value: <0.001), or are not married—including those who have a steady partner (OR: 2.6; 95%CI: 2.0–3.4; p-value: <0.001), live with someone (OR: 1.5, 95%CI: 1.2–1.9; p-value: 0.003) or are divorced but not widowed (OR: 1.8; 95%CI: 1.1–2.9; p-value: 0.014), compared to married women. The number of children a woman had steadily decreased her OR of being high risk HPV positive. Other factors such as age at first sexual intercourse, the use of contraceptives and income level did not show high evidence for an association with HPV positivity in univariate analysis.

**Table 3:** Univariate logistic regression analysis of potential risk factors for high risk HPV positivity.

Characteristic	Total	High risk HPV+	OR	95% CI	p-value
**Age**					**<0.001**
18–24	545	227	Ref.		
25–34	727	218	0.6	0.5–0.8	**<0.001**
35–44	392	110	0.6	0.4–0.7	**<0.001**
45–54	203	51	0.5	0.3–0.7	**<0.001**
55–65	76	22	0.6	0.3–1.0	**0.036**
**Education**					
None	335	91	Ref.		
Primary	427	151	1.5	1.1–2.0	**0.016**
Junior High School	784	248	1.2	0.9–1.7	0.137
Secondary	248	97	1.7	1.2–2.5	**0.002**
Post Secondary	72	24	1.3	0.8–2.3	0.293
Post Graduate	62	15	0.9	0.5–1.6	0.627
**Income level per month**					**0.019**
<100 GH¢	976	317	Ref.		
100–250 GH¢	321	87	0.8	0.6–1.0	0.072
251–500 GH¢	100	23	0.6	0.4–1.0	0.054
>500 GH¢	106	28	0.8	0.5–1.2	0.204
**Occupation**					
Apprentice/Student	183	80	Ref.		
Farmer	487	125	0.4	0.3–0.6	**<0.001**
Hairdressing	82	29	0.7	0.4–1.2	0.203
Health care	34	8	0.4	0.2–0.9	**0.032**
Seamstress	110	44	0.9	0.5–1.4	0.533
Teacher	73	19	0.5	0.3–0.8	**0.010**
Trader	654	201	0.6	0.4–0.8	**0.001**
Unemployed/House wife/Retired	127	47	0.8	0.5–1.2	0.238
Food vendor	43	15	0.7	0.3–1.4	0.293
Office	29	10	0.7	0.3–1.5	0.352
Cleaner	9	2	0.4	0.1–1.8	0.220
**Marital Status**					
Single	174	77	2.6	1.8–3.6	**<0.001**
Have a steady partner	387	174	2.6	2.0–3.4	**<0.001**
Living with someone (unmarried)	415	132	1.5	1.2–1.9	**0.003**
Married	825	196	Ref.		
Divorced	83	30	1.8	1.1–2.9	**0.014**
Widowed	58	19	1.6	0.9–2.8	0.125
**Number of sexual partners**					**0.003**
None	23	5	Ref.		
1	750	224	1.5	0.6–4.2	0.404
2–3	938	306	1.7	0.6–4.7	0.276
>3	208	85	2.5	0.9–7.0	0.082
**Number of children**					**<0.001**
None	342	148	Ref.		
1–2	745	250	0.7	0.5–0.9	**0.002**
3–4	476	138	0.5	0.4–0.7	**<0.001**
5–6	240	61	0.5	0.3–0.6	**<0.001**
>6	135	29	0.4	0.2–0.6	**<0.001**
**Age at first intercourse**					**0.045**
<15	64	26	Ref.		
15–18	935	319	0.8	0.5–1.3	0.291
19–22	608	188	0.7	0.4–1.1	0.115
23–26	103	31	0.6	0.3–1.2	0.164
>26	24	6	0.5	0.2–1.4	0.179
**Contraceptive Use**					
None	1278	397	Ref.		
Injectable	341	123	1.3	1.0–1.6	0.079
Pill	138	45	1.1	0.7–1.6	0.710
Norplant/Jadelle	81	33	1.5	1.0–2.4	0.071
Condom	65	19	0.9	0.5–1.6	0.755
Abstinence	24	7	0.9	0.4–2.2	0.842
IUCD	4	0			
Other	8	3	1.3	0.3–5.6	0.696
**Current smoking**					
Yes	17	5	0.9	0.3–2.5	0.797
No	1917	620	Ref.		
**Usage of herbal vaginal preparation**					
Yes	246	74	0.9	0.7–1.2	0.422
No	1691	552	Ref.		

Abbreviations: OR—odds ratio; CI—Confidence interval; HPV—Human Papillomavirus; GHC—Ghana Cedi; IUCD—Intrauterine Contraceptive Device

After multivariate logistical regression only the factors age, marital status and number of sexual partners appeared to have a high association for infection (Table 4). Here, as mentioned above younger age had a higher OR for infection. Being single (AOR: 2.6; 95%CI: 1.8–3.8; p-value: <0.001), having a steady partner but not living together (AOR: 2.2; 95%CI: 1.6–2.9; p-value: <0.001) and being divorced (AOR: 1.9; 95%CI: 1.2–3.0; p-value: 0.011) were highly associated with high risk HPV positivity. Living with someone but not being married (AOR: 1.3; 95%CI: 1.0–1.7; p-value: 0.047) was also associated with infection. The more sexual partners a woman had the higher the ORs for infections were (e.g. >3 partners AOR: 5.0; 95%CI: 1.7–14.6; p-value: 0.004). Interestingly, there was already a high association for infection with the first sexual partner (AOR: 3.3; 95%CI: 1.1–9.3; p-value: 0.027).

**Table 4:** Risk factors associated with high risk HPV positivity from multivariate analysis.

	High risk HPV positivity			Multiple high risk HPV type positivity		
Characteristic	Adjusted OR	95% CI	p-value	Adjusted OR	95% CI	p-value
**Age**						
18–24	Ref.			Ref.		
25–34	0.7	0.5–0.8	**0.001**	0.7	0.5–1.0	0.083
35–44	0.7	0.5–0.9	**0.013**	0.4	0.2–0.7	**0.001**
45–54	0.6	0.4–0.8	**0.005**	0.7	0.4–1.3	0.243
55–65	0.7	0.4–1.2	0.172	0.6	0.3–1.6	0.331
**Marital Status**						
Single	2.6	1.8–3.8	**<0.001**	2.5	1.4–4.4	**0.002**
Have a steady partner	2.2	1.6–2.9	**<0.001**	2.5	1.6–3.9	**<0.001**
Living with someone (unmarried)	1.3	1.0–1.7	**0.047**	1.8	1.1–2.8	**0.015**
Married	Ref.			Ref.		
Divorced	1.9	1.2–3.0	**0.011**	3.5	1.8–7.0	**<0.001**
Widowed	1.6	0.9–2.9	0.135	1.4	0.5–4.1	0.579
**Number of sexual partners**						
None	Ref.			-		
1	3.3	1.1–9.3	**0.027**	Ref.		
2–3	3.7	1.3–10.4	**0.016**	1.1	0.8–1.5	0.675
>3	5.0	1.7–14.6	**0.004**	1.7	1.1–2.8	**0.028**

Abbreviations: OR—odds ratio; CI—Confidence interval

## Discussion

Sample collection via self-sampling worked exceptionally well in this study. The integration of recruitment and sampling in the responsibilities of CHW system allowed simultaneous screening across the District within only 5 weeks. With 98% of the samples providing valid HPV genotyping results, this sampling method should be considered for large-scale screening programs, especially for widespread communities.

### HPV prevalence in Ghana

The results of this cross-sectional HPV prevalence study show an exceptionally high prevalence of the high risk HPV genotypes of 32.3%. This is a 52% increase compared to the initially expected prevalence of 21.3%, which had been used as an estimate for Western Africa in the sample size calculation [15]. Even among women aged 30–49 years, the WHO recommended screening age, high risk HPV prevalence is high and above the estimate for Western Africa with 27.3%.

Looking at some of the other scarce studies conducted among the general population in Ghana, a prevalence of 10.7% was found among 75 women (mean age: 33.3 years) attending the gynecology outpatient clinic in Accra [11], or of 42% among 100 HIV negative women (mean age: 40.9 years) attending the outpatient department of a referral hospital in Kumasi [12]. Schulze et al. reported a prevalence of 13.9% for high risk HPV among pregnant women in Eikwe, Southwest Ghana [13]. Although not directly reflecting the situation in Ghana, the extensive meta-analysis including 1 million women across 5 continents estimates the prevalence for Western Africa at 19.6% after adjusting for various factors such as geographical subregion, mean age of women, HPV testing method etc. [21]. A study from the neighboring country Burkina Faso reports a prevalence similar to our findings of 34.4% among women aged 35.5 years recruited among visitors of an urban medical center [22]. One could hypothesize that women referred to and recruited from a medical center possibly with characteristic symptoms have a higher risk of being HPV positive, may rather be considered a referral population and that this reported prevalence is therefore an overestimation. However, our findings support such high prevalence also to be seen among the general population. This is further supported by a different study conducted in Western Burkina Faso finding a prevalence of 38.3% [23] and a prevalence of 33.2% reported from Benin [24]. Other studies report prevalence or type distribution among selected populations, such as women with cervical lesions or even cervical cancer or HIV-positive women, which cannot be compared to this prevalence among the general population. Ghana has an overall low HIV rate of 1.5% and even though the Volta Region with 2.7% has the highest reported HIV prevalence within the country, it is still low compared to other Sub-Saharan African countries and may not explain the high prevalence found [25]. While the prevalence seen here may not be representative for the prevalence in Western Africa or even other regions in Ghana, given the large sample size it is a valid representation of the prevalence in the North Tongu District of Ghana, which is higher than so far reported and expected.

Nevertheless, the majority of women (65.4%) recruited for this study was at the age of 18–34 years with a prevalence of 35.0% for this age group. Looking at the age distribution of the population living in North Tongu district from the 2010 Population and Housing Census, the age group 20–34 years comprises about 51.0% among the 20–64 years old women (similar to the age range meeting the study inclusion criteria) of the North Tongu population and is hence overrepresented among the women recruited for this study. On the other hand the older age group (45–65 years; 34.6% of the study participants) comprises 48.9% among 20–64 years old in North Tongu women’s population and is therefore underrepresented here. Since the peak of HPV infection is expected among women aged ≤25 years [26], this could cause an overestimation of the actual prevalence among the general population in the North Tongu District.

Additionally, it has been described that although the accuracy of HPV testing from self-collected samples compared to physician-obtained samples is similar, there is a tendency of more HPV types being detected in self-collected samples from the cervicovaginal compartment as compared to physician-collected cervical-targeted sampling [27–30]. This could also lead to an overestimation of the prevalence described here, compared to commonly reported cervical HPV prevalence. Another methodological factor that could influence the reported prevalence of various studies is the sensitivity of testing methods for HPV detection. Different HPV detection methods have slightly different sensitivities and would therefore over- or underestimate the true HPV prevalence in the population.

### HPV type distribution

The five most prevalent high risk HPV types found in this study are 16, 52, 35, 59 and 56, in descending order, known to contribute to a total of 59.4% of cervical cancer cases in the African Region, as defined by WHO [31]. The types 35, 56 and 59 are not included in any HPV vaccine, but in this study contribute to a prevalence of 12.4% (some women are positive for two of the mentioned types). Looking at the prevalence of these types in invasive cervical cancer, as reported for Western Africa, all three types are among the top 10 cervical cancer causing HPV types, being prevalent in 16.4% of cancers, with HPV59 alone contributing almost 10% [31]. The prevalence of these non-vaccine covered HPV types shows that certain HPV vaccines may not be as effective in this region as compared to other regions of the world. The prevalence of distinct HPV genotypes varies between world regions and also in the African continent. Reasons could be e.g. the local prevalence of HIV that leads to more multiple and persistent infections by other genotypes than HPV16. Data on prevalence from sufficiently large studies are sparse for Western Africa and a direct comparison cannot be drawn due to use of different HPV genotyping tests. However, HPV35 (rank 4–5) and 39 (rank 10) have shown a dominating proportion in other African regions [31].

### Multiple HPV infections

9.7% of the women in this investigated population had multiple high risk HPV infections. Multiple high risk HPV infections are often reported among HIV positive persons [32–34]. However, it may not fully explain the results seen from the population recruited for this study. As mentioned above, Ghana has an overall low HIV rate, which therefore may not be the only contributor to the high prevalence of multiple high risk infections [25]. Another aspect described to be associated with high prevalence of multiple infections is high promiscuity among sex workers [35]. Among the women recruited 10.6% had more than 3 sexual partners and the mean number of partners is 2.1. The number of sexual partners was associated with single high risk infection, but also with multiple high risk HPV infection from multivariable regression analysis of this study (Table 4). Multiple type positivity is an important factor to investigate, as it increases somebodies risk of infection with additional HPV types and is under debate to also reduce their risk to clear prevalent types, meaning prevalent types persist longer [36]. It also reduces the survival of cervical cancer patients and has a higher rate of distant tumor recurrences [37].

### Risk factors for HPV positivity

Based on the self-reported answers provided to the questionnaires several risk factors have been investigated and shown to be associated with high risk HPV infection. Knowledge of these risk factors is of great importance to target women at highest risk of infection with screening programs, especially in countries with limited resources for screening. Three risk factors remained associated with high risk HPV infection after multivariable regression analysis: Age, relationship status, and number of sexual partners.

Transmission of genital HPV and hence infection occurs with the onset of sexual activity and HPV infection has been described to peak among younger women aged ≤25 years [26]. This is supported by the results from this study with the highest HPV prevalence of 41.7% found among women younger than 25 years. The multivariate analysis further confirmed this, showing the lowest ORs among older women compared to women younger than 25 years.

Being single or divorced was highly associated with infection compared to being married. Surprisingly, having a steady partner and living with someone but not being married was also associated with infection, even after adjusting for age and the number of sexual partners. This might be due to ambivalent interpretation of these two categories by the study participants. The questionnaires were filled out under supervision of the CHW and that might have influenced the answers given. Possibly different interpretation of relationship length influenced the category of “having a steady partner”. Other studies reported that being in a relationship for 12 months or longer is protective for infection, indicating that the duration of a relationship seems to be an important factor [38]. In the literature early age at marriage is also described to be associated with HPV infection [39], which is a factor that was not asked for. Beside from this, the number of sexual partners in a lifetime is one of the most important factors associated with HPV positivity. Various studies could show, similarly to our findings, that an increasing number of sexual partners also increase the OR of being high risk HPV positive [39–42]. While other studies mostly focus on the number of sexual partners as a risk factor and rarely investigate the relationship status, interestingly we see both factors to be independent risk factors. Our results show an association between relationship status and the number of sexual partners, but when including both in the multivariable regression analysis, both remain independently highly associated with HPV infection. This means that both are independent risk factors despite their association. This has not yet been investigated in other studies.

Other studies found lower levels of formal education to be associated with infection [43]. In our population we can only partially confirm this, since we found primary and secondary education to be associated with higher risk of infection, however we did not see a difference in association between no or post-graduate education. Educational level was also not associated anymore with high risk infection in the multivariate analysis after adjustment. Also, cigarette smoking has been reported to be associated with HPV infection [41]. Since only about 1% of our study participants indicated to smoke we could not assess this risk factor.

## Limitations

While this cross-sectional study is sufficiently powered to detect a difference in HPV prevalence compared to the estimate stated for Western Africa, it was not meant to be powered to assess risk factors. The sociodemographic and behavioral characteristics are based on self-reported questionnaires and may therefore be subject to biased answers. The associations we found have to be investigated in larger studies and a similar context.

Additionally, problems with sample genotyping resulted in longer waiting times than expected for the women participating. Since women may travel across the country with the harvest season and also the CHWs responsible for the respective area change locations for training, to mention only a few potential factors, only about half of the positively tested women travelled to the clinic for follow-up. The unexpectedly high prevalence made it difficult for all women to receive colposcopy and therefore the additional cytology triage step was introduced. Therefore, we cannot make any conclusions on the clinical status of all women tested positive and hence excluded the clinical outcome from any further analysis.

## Conclusion

The main finding of this study is a higher prevalence of high risk HPV than expected until now. Secondly, we found that three HPV types, namely HPV35, 56 and 59, are among the top 5 types found but not covered by any HPV vaccine even though they are found in 16.4% of cervical cancers in Western Africa [31]. Thirdly, risk factors found for HPV positivity were young age, the number of sexual partners and the relationship status.

Despite the high HPV prevalence found in the investigated population there is currently no national screening program for cervical cancer screening in Ghana. Although the recent national control program for non-communicable diseases (NCDs) states an instituted cervical cancer screening system as a national policy priority, little of the suggested means have been implemented until today [44] and the cervical cancer screening coverage remains low with 2.8% of women aged 25–64 years [45]. This is further highlighted by an analysis including cervical cancer patients from the Catholic Hospital Battor in Ghana, showing that late presentation at the clinic was the main risk factor for cervical cancer diagnosis [5]. The high prevalence of non-vaccine preventable HPV types shows the importance of secondary prevention efforts, even if primary prevention with the available vaccines would be fully implemented. The results of this study can therefore help in tailoring a screening system that is adapted to a higher than expected HPV prevalence.

Self-sampling worked well and provided adequate samples for HPV-based screening. However, the high HPV prevalence found would require further triage and follow-up for a large number of women, which can easily overburden the health system. This is a major aspect when deciding on the feasible cervical cancer screening algorithm introduced on a wider scale.

## Supporting information

S1 File Questionnaire. (PDF)

S1 Fig Flow chart of clinical follow up. (TIF)
