# Supplementary material for: Characterization of Human Papillomavirus prevalence and risk factors to guide cervical cancer screening in the North Tongu District, Ghana
Source: PLoS One. 2019 Jun 27;14(6):e0218762. doi: 10.1371/journal.pone.0218762 (PMC6597158; doi:10.1371/journal.pone.0218762)
Supplement: S1 File — (PDF) [file pone.0218762.s001.pdf]

Rural Cervical Cancer Program

Prevalence of HPV infection in the North Tongu District of Ghana

1. Hospital card no.	
2. Study ID no. (Use Stickers)	
3. Initials of patient (Surname first)	__ __ __
4. Date of attendance	____/____/____ (*DD/MM/YY*)
5. District	
6. Village	
7. Where sample was taken: **1**-Clinic **2**-CHPS Compound **3**-Home	
8. Who took the sample **1**-Self-Unsupervised **2**-Self-Supervised **3**-Health worker	

A. Socio-demographic characteristic

1. Age of patient __ __	yrs
2. Do you normally reside in this district? (**1**-Yes, **2**-No)<br>2b. If "No" please specify current place of residence ______________________________	__
3. Highest level of education completed<br>(**0**-None, **1**-Primary 2. Junior High School **3**-Secondary, **4**-Post-secondary, **5**-Post-graduate)	
4. Do you earn any income? 1-Yes 2-No	
5. *If "Yes" please specify level of income per month*<br>(**1**- <100GH¢, **2**-Between 100 – 250Gh¢, **3** – Between 251-500Gh¢, **4** – Above 500Gh¢)	
6. How do you normally pay your medical bills?<br>(**1**-Self i.e. from personal income, **2** – Paid for or assisted by relatives, **3**-My present/former employer,<br>**4** – National Health insurance, **5**-Other: please specify:____________________)	
7. Do you currently smoke **1**- Yes **2**-No<br>7a. If you currently smoke, how many cigarettes do you smoke per day?<br>7b. If you do not currently smoke, did you smoke in the past? **1**- Yes **2**-No	
8. How many pregnancies have you had in the past? ____________<br>8a. How many of these pregnancies resulted in stillbirth? ____________<br>8b. How many of these pregnancies resulted in miscarriage/abortion? ____________	
9. How many children do you have? ____________	
10. Marital status<br>(**1**- married, **2**- single, never married, **3** – widow, **4** – divorced,/separated, **5** – Other, please specify:__________)	__
10a. If married/widowed/ divorced/ separated, age at first sexual intercourse ________ (yrs)<br>10b If single, have you ever had sexual intercourse before? (**1**-Yes **2**-No)<br>10c. *If yes, Age at first sexual intercourse* __________________	
If you ever had sexual intercourse before, please continue with the following questions, otherwise continue with part B.	
11. How many sexual partners have you had? ___________________	
12. Any sexual partner had more than one partner during lifetime **1**-Yes **2**-No **3**-Don't know	
13. What is your current contraceptive?<br>**0**-None **1**-Pill **2**-Injectable **3**-Condom 4-Norplant/Jadelle **5**-IUCD **6**-Other (Specify______)	
14. Do you use a vaginal herbal preparation? **1**-Yes **2**-No	

B. Medical history

1. Have you ever been diagnosed with any of the following?	1-Yes	2-No	3-DK
i. Hypertension	_____	_____	_____
ii. Diabetes	_____	_____	_____
iii. Asthma	_____	_____	_____
iv. Sickle-cell	_____	_____	_____
v. Cancer	_____	_____	_____
vi. HIV/AIDS	_____	_____	_____
vii. Other 1 (please specify_____________________)	_____	_____	_____

If you have HIV/AIDS,
1a. How long has it been since diagnosis (in years and/or months)? _____/______(yrs/mnths)
1b. Are you on antiretroviral medication?
__1__-Yes; for how long? ______/____(yrs/months) __2__-No __3__-Don't Know
1c. Are you symptomatic? __1__-Yes __2__-No __3__-Don't Know

2a. Last menstrual period [LMP] _____/___/__ ___ (*DD/MM/YY*)
2b. Interval since LMP
1-Less than 4 months **2**- 4 to 12 months **3**- More than 12 months **4**-Does not remember

4. Post-coital spotting or bleeding **1**-Yes **2**-No

5. Do you have or ever had:
1-Pelvic pain with discharge **2**-Abnormal offensive discharge **3**-Ulcer/sore **4**-Warts **5**-None of these

6. Current partner/spouse ever had
1- Pain on urination **2**-Abnormal offensive discharge **3**-ulcer/sore **4**-Warts **5**-None of these **6**- Don't Know

7. Former partner/spouse ever had
1- Pain on urination **2**-Abnormal offensive discharge **3**-Ulcer/Sore **4**-Warts **5**-None of these **6**-Don't Know **7-**Not applicable

8. Mother or Sister has or had cervical cancer **1**-Yes **2**-No **3**-Don't know

9. Have you ever been screened for cervical pre(cancer)? **1**- Yes **2**-No **3**-Don't know.

If Yes;
9a.*How many times have you been screened in the past? ----- times*
9b. When were you last screened? ____/___/___(DD/MM/YY)
9c. Have you ever had an abnormal pap smear? __1__- Yes; At the last screening? yes/no
__2__-No
__3__-Don't know
9d Have you ever had a biopsy? __1__- Yes; please briefly state the biopsy results___________________
__2__-No
__3__-Don't know

C. Presenting complaints (Briefly describe) [Applies only to patients screened in hospital]

**Specific symptoms**	Duration in months				Severity grade*			
	**<1**	**1-3**	**4-6**	**>6**	**1**	**2**	**3**	**4**
i. Bleeding Per Vaginam	___	___	___	___	_	_	_	_
ii. Painful intercourse	___	___	___	___	_	_	_	_
iii. Post-coital bleeding	___	___	___	___	_	_	_	_
iv. other:________________________	___	___	___	___	_	_	_	_

*Severity grade: **1 - Mild** i.e. present with no effect on normal activities, **2- Moderate** i.e. minimal tolerable effect (symptomatic relief would suffice), **3- Severe** i.e. having significant effect on normal activity, **4-Serious** i.e. incapacitating, requiring hospitalization or potentially life-threatening.

D. Cervical cancer awareness

1. Have you heard about Cervical Cancer? *(**1**-Yes, **2**-No)*	__
1a. If Yes, how did you hear about<br>**1** -Was just told about it, **2** -In this hospital **3** -Radio/TV **4** -Other (Please sp:_______)<br>**5**-Not applicable	
1b. If Yes, how long ago did you hear about it?<br>***1-** Less than 6 months **2 -** Between 6 to 12 months **3-**More than 12 months **4**-Not applicable*	
2. If there were facilities for screening for early detection, would you come regularly for check-up?<br>***1**-Yes, **2**-No*	
3. *If Yes, how much will you be willing to pay for such a service?*<br>**1**- <10GH¢, **2**-Between 10 - <20Gh¢, **3** - Between 20 - <30Gh¢, **4** – at least 30Gh¢	___
4. Is your partner/husband aware of Cervical Cancer? **1**-Yes **2**-No **3**-Don't Know	___
5. Is your partner/husband aware that you are coming for screening for Cervical Cancer?<br>**1**-Yes **2**-No **3**-Don't Know	
6. Does your partner/husband approve of your undergoing this Cervical Cancer screening?<br>**1**-Yes **2**-No **3**-Don't Know	

E. Acceptability of DELPHI Screener

1. If you took the sample by yourself, please indicate how easy or difficult it was to use the DELPHI screener for self-sampling?<br>**1**-Very Easy **2**-Easy 3-Difficult **4**-Very difficult
2. If you took the sample by yourself, please indicate how comfortable you felt collecting your own sample with the Screener?<br>**1**-Very Comfortable **2**-Somewhat comfortable **3**-Somewhat uncomfortable **4**-Very uncomfortable **5**-not applicable
3. If the sample was taken by a health worker, how comfortable was it?<br>**1**-Very Comfortable **2**-Somewhat comfortable **3**-Somewhat uncomfortable **4**-Very uncomfortable **5**-Not applicable
4. Prior to this screening, had a health professional ever taken your sample during a pelvic examination?<br>**1**-Yes **2**-No<br><br>4b. *If you answered "Yes",* how comfortable did you feel when the health professional collected your samples at your last pelvic exam?<br>**1**-Very Comfortable **2**-Somewhat comfortable **3**-Somewhat uncomfortable **4**-Very uncomfortable **5**-Don't remember
6. If the Screener works as well as going to the doctor, would you get checked more often, less often or about the same?<br>**1**-More often **2**-The same **3**-Less often.
7. If both sampling by brush and by DELPHI Screener can determine your risk of cervical cancer equally, which one would you prefer?<br>**1**-Sampling with speculum and brush **2**-Sampling with the self sampler/vaginal lavage **3**-Not applicable

_________________ ________

Signature Date
