# Supplementary figures and images for: Characterization of Human Papillomavirus prevalence and risk factors to guide cervical cancer screening in the North Tongu District, Ghana
Source: PLoS One. 2019 Jun 27;14(6):e0218762. doi: 10.1371/journal.pone.0218762 (PMC6597158; doi:10.1371/journal.pone.0218762)

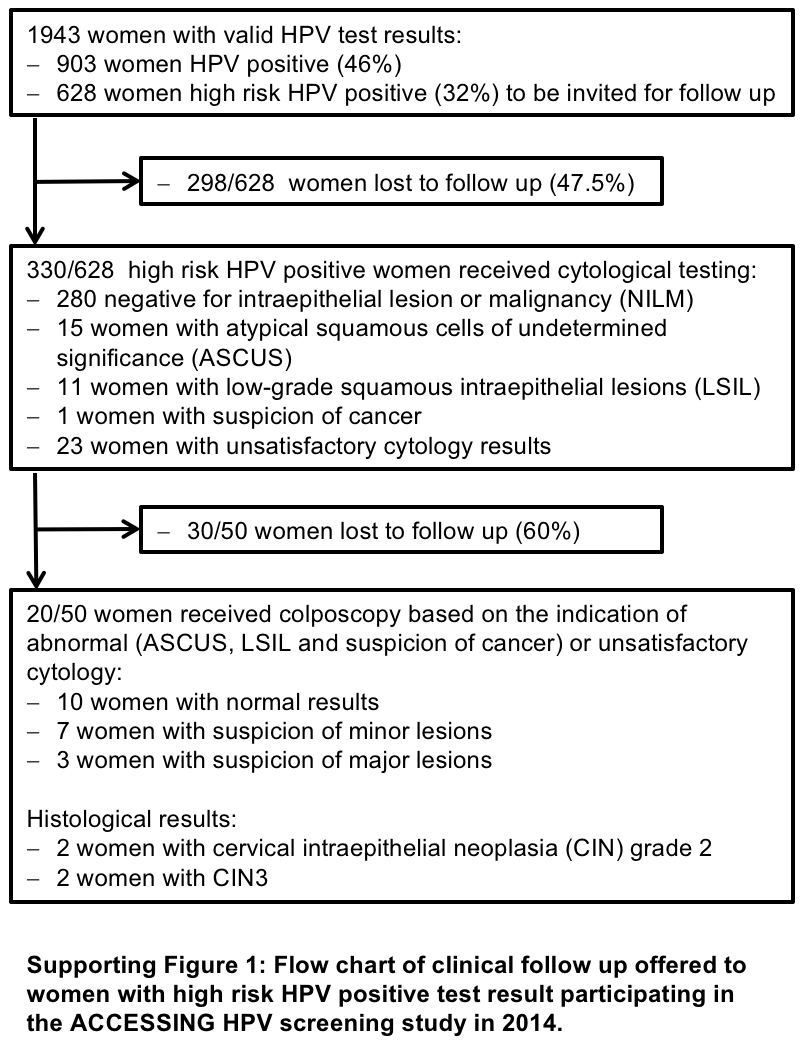

Supplement: S1 Fig — (TIF) [file pone.0218762.s002.tif]
